# Antimicrobial usage in pig production: Effects on *Escherichia coli* virulence profiles and antimicrobial resistance

**DOI:** 10.4102/ojvr.v86i1.1743

**Published:** 2019-10-31

**Authors:** Rukayya H. Abubakar, Evelyn Madoroba, Oluwawemimo Adebowale, Olubunmi G. Fasanmi, Folorunso O. Fasina

**Affiliations:** 1 Department of Veterinary Tropical Diseases, Faculty of Veterinary Sciences, University of Pretoria, Pretoria, South Africa; 2 Department of Biochemistry and Microbiology, University of Zululand, KwaDlangezwa, South Africa; 3 Department of Veterinary Microbiology and Parasitology, College of Veterinary Medicine, Federal University of Agriculture, Abeokuta, Nigeria; 4 Department of Production Animal Studies, Faculty of Veterinary Sciences, University of Pretoria, Pretoria, South Africa; 5 Department of Animal Health, Federal College of Animal Health and Production Technology, Ibadan, Nigeria; 6 Emergency Centre for Transboundary Animal Diseases, Food and Agriculture Organisation of the United Nations, Dar es Salaam, Tanzania

**Keywords:** antimicrobial, *Escherichia coli*, microbial drug resistance, virulence

## Abstract

Antimicrobials (AM) are used for growth promotion and therapy in pig production. Its misuse has led to the development of resistant organisms. We evaluated *Escherichia coli* virulence genes, and compared phenotypic–genotypic antimicrobial resistance (AMR) patterns of faecal *E. coli* from pigs receiving routine farm treatment without antimicrobial agents against pigs treated routinely with AM over 70 days. Recovered *E. coli* were tested for AMR using disk diffusion and polymerase chain reaction. Virulence genes were detected in 24.8% of isolates from antimicrobial group and 43.5% from non-antimicrobial group (*p* = 0.002). The proportion of virulence genes heat-stable enterotoxins a & b (STa, STb), enteroaggregative heat stable enterotoxin 1 [EAST1] and Shiga toxin type 2e [Stx2e]) were 18.1%, 0.0%, 78.7% and 3.0% for antimicrobial group and 14.8%, 8.5%, 85.1% and 12.7% for non-antimicrobial groups, respectively. Resistance to oxytetracycline was most common (*p* = 0.03) in samples collected between days 10 and 21. Resistance shifted to amoxicillin on days 56–70, and trimethoprim resistance was observed throughout. Seventeen phenotypic AMR combinations were observed and eight were multidrug resistant. At least one tetracycline resistance gene was found in 63.9% of the isolates. tet (A) (23.3%) was most common in the antimicrobial group, whereas tet (B) (43.5%) was prevalent in the non-antimicrobial group. Usage or non-usage of antimicrobial agents in growing pigs does not preclude virulence genes development and other complex factors may be involved as previously described. Heavily used AM correspond to the degree of resistance and tetracycline resistance genes were detected during the growth phase.

## Introduction

*Escherichia coli* is a major cause of diarrhoea in pigs (piglets and weaners) at different levels of intensity worldwide (Vu Khac et al. 2006). In piglets, *E. coli* diarrhoea may be followed by terminal septicaemia, which is an important cause of economic loss for pig producers globally (Toledo et al. 2012). The estimated pig population in South Africa as of 2010 was about 1.5 million (Meissner, Scholtz & Palmer 2013), while the population worldwide is about 1 billion. Pork serves as an important source of protein for human beings in developing countries or areas where pork consumption is not prohibited (Madzimure et al. 2012).

Diarrhoeagenic *E. coli* pathovars involved in pig enteric infections include mainly enterotoxigenic *E. coli* (ETEC) encoding heat-stable enterotoxins a & b (STa, STb), enteroaggregative heat stable enterotoxin 1 [EAST1]) and/or heat-labile (LT) enterotoxins, causing secretory diarrhoea in newborn and weaned piglets (Gyles & Fairbrother 2010). In addition, Shiga toxin *E. coli* (STEC) strains encode the Shiga toxin type 2e (Stx2e) that causes oedema disease but not diarrhoea (MacLeod, Gyles & Wilcock 1991). Interestingly, some strains harbour both the Stx2e genes and enterotoxin genes capable of causing symptoms of both oedema disease and diarrhoea in the same animal (STEC/ETEC) (Barth, Schwanitz & Bauerfeind 2011). Many porcine ETEC and STEC strains have fimbrial structures on their surface that like LT, STa and STb enterotoxins are usually plasmid mediated (Dubreuil, Isaacson & Schifferli 2016). These fimbriae are termed colonisation antigens and they enable the bacteria to colonise the epithelial surface of the pig’s small intestine, namely, F4 (K88), F5 (K99), F6 (P987), F18 and F41 usually found in pig ETEC (Blanco et al. 2006). Antimicrobial agents are frequently used in the treatment and control of these enteric infections in pigs.

A recent study has shown that administration of antimicrobial agents increases the risk of antimicrobial resistance (AMR) (Burow et al. 2014). Other factors like stress from temperature, crowding and management also seem to contribute to the occurrence of AMR in animals (Sørum & Sunde 2001). The commensal bacteria in animals may become a reservoir of resistance to genes for pathogenic bacteria. This may contaminate meat and meat products meant for human consumption (Van den Bogaard & Stobberingh 2000). Recent reports have indicated that the prevalence of antimicrobial-resistant *E. coli* is on the increase (Luppi et al. 2015; Toledo et al. 2012) and the infections caused by the resistant bacteria usually fail to respond to treatment by specific antimicrobial agents (Rice 2009). This may be associated with the increased proliferation of bacterial pathogens, re-infection rates, chronicity, opportunistic infections with resistant organisms and a reduced life span (Capita & Alonso-Calleja 2013).

Resistance to tetracycline determined phenotypically has been reported more frequently among bacteria isolated from pigs than previously known (Tadesse 2012). The resistance is known to be inducible and occurs basically because of the acquisition of tetracycline (tet) or oxytetracycline (otr) genes (Roberts 2011) and many isolates from pigs have shown multidrug resistance genes located on plasmids (Lutz et al. 2011).

*Escherichia coli* infections have been identified to be a challenge in the South African pig production industry (Fasina, Bwala & Madoroba 2015; Kanengoni et al. 2017). A recent study showed that the prevalence of ETEC, STEC and EAST1 and associated fimbrial genes in indigenous South African breeds was high (Mohlatlole et al. 2013), an outbreak of multidrug resistance coliceptisaemia in weanling pigs was reported (Ikwap et al. 2016) and an investigation on piglet mortality in a farm was characterised to be associated with STEC (Kanengoni et al. 2017). Treatment and control of disease outbreaks in the South African pig industry involves the use of antimicrobial agents (Henton et al. 2011). The purpose of this investigation was to determine the effect of antimicrobial treatment on the prevalence of virulence genes and AMR in intestinal *E. coli* in growing pigs.

## Methodology

### Approvals, animal care and welfare

**FIGURE 1:**
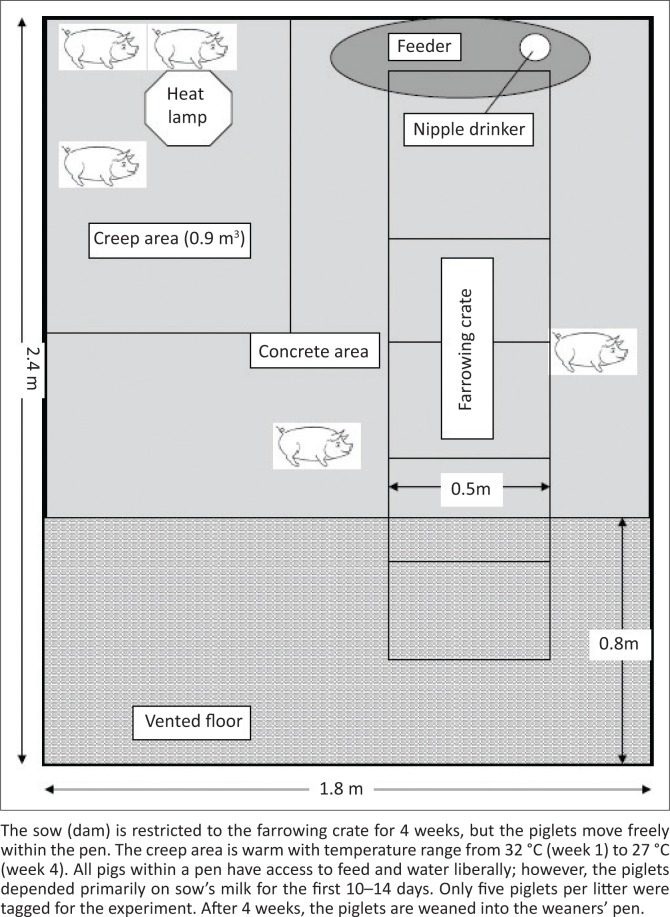
Standard farrowing pen with creep area, farrowing crate, concrete and vented floor.

All pigs involved in the study were placed under a 24-hour monitoring programme conducted by the pig farm team (attendant and manager) for the duration of the study using the assessment and control of the severity of scientific procedures on laboratory animals scoring system and the guide to defining and implementing protocols for the welfare assessment of laboratory animals (Hawkins et al. 2011; Wallace et al. 1990). All piglets were housed in the farrowing unit with crates, creep area, heating lamps and unlimited access to the dam’s teats, creep feed and water *ad libitum* (Figure 1). A total of 4 out of 10 piglets were removed in the last 2 weeks of the study because of laboratory-confirmed colisepticaemia (oedema disease). For each animal to be removed by euthanasia (carbon dioxide asphyxiation in piglets or humane slaughter in weaners or growers) or sudden death, the humane endpoint was set with a Severity Index (SI) score of > 20 on the Laboratory Animal Science Association (LASA) Working Party Scale (Wallace et al. 1990) and/or a score of **≥** 6 on the BVAAWF/FRAME/RSPCA/UFAW Joint Working Group Scale (Hawkins et al. 2011). No other unexpected occurrence was recorded in the course of the experiment. The situation that triggered the scores for removal includes pain incompatible with animal welfare like prostration, nervous manifestation that affected normal movement and loss of the ability to ingest food for 24–48 hours.

### Study design

A small-scale commercial pig farm was identified in the Gauteng province of South Africa, and two pregnant sows were monitored clinically and physiologically until the day of farrowing. Piglets (*n* = 10) were randomly selected (five from each sow together with their unselected litter mates) and placed into two groups. All 10 selected piglets were tagged into groups A (non-antimicrobial group: with five tagged piglets and other non-tagged litter mates that were kept in one farrowing pen under routine farm management practices but without any form of antimicrobial usage) and B (antimicrobial group: with five piglets and other non-tagged litter mates that were kept under the routine management practices of the farm, which included administration of multivitamins, deworming, tail docking, vaccination, provision of warmth and antimicrobial administration to the sick animals). Effort was made to ensure the prevention of cross-contamination from the environment and between the groups by leaving three farrowing pens vacant (5.4 m width) between the two groups ensuring caretakers attend to the non-antimicrobial group before the antimicrobial group daily.

### Sample collection

Rectal swabs were taken from all 10 piglets (four swabs per animal at each collection) with a sterile swab stick and each swab labelled with the specific pig identification number and age (days), and transported to the Agricultural Research Council-Onderstepoort Veterinary Research Feed and Food Analysis Laboratory (Bacteriology section) on ice. All samples were processed in the laboratory within 2 h of sample collection. The samples were collected periodically on days 1, 5, 10, 21, 28, 35, 56 and 70 from all pigs.

### Classical microbiological analysis

#### Escherichia coli isolation and antimicrobial resistance testing

The swabs were streaked directly on MacConkey agar (Oxoid, Basingstoke, United Kingdom [UK]) plates and incubated aerobically overnight at 37 °C. Lactose fermenting colonies (*n* = 4–6) were selected and sub-cultured on MacConkey agar. The pure colonies were then transferred to nutrient agar (Oxoid, Basingstoke, UK) plates. The isolates on nutrient agar plates were subjected to an indole test together with other biochemical reactions for *E. coli* identification. For this purpose, 10 mL of tryptone water was inoculated with pure culture and incubated over night at 37 °C. Kovacs reagent (one to two drops) was added and the formation of a red ring was indicative of *E. coli*. In addition, an Indole test, Methyl red test, Voges-Proskauer test and a Citrate utilization test (IMViC) were also conducted. Subcultures were also cultured on 5% sheep blood agar to determine the haemolytic characteristics of the *E. coli. Escherichia coli* ATCC 25922 and *E. coli* O157 were used as controls. Antimicrobial susceptibility testing was done using the Kirby–Bauer disk diffusion method. The zones were interpreted according to the Clinical Laboratory Standards Institute (CLSI) guidelines (Clinical Laboratory Standards Institute [CLSI] 2015).

The following antimicrobial agent discs were selected according to standard regulations (CLSI 2015; Food and Drug Administration 2012; World Health Organization 2016): amoxicillin (AML) 10 *µ*g; cefotaxime (CTX) 30 *µ*g; oxytetracycline (OT) 30 *µ*g; kanamycin (K) 30 *µ*g; florfenicol (FFC) 30 *µ*g; enrofloxacin (ENR) 5 *µ*g and trimethoprim (W) 5 *µ*g (Table 1). The criteria that were used for selecting the antimicrobial agents are the use on the farm during pig production and the recommendations for testing of bacteria from animals (CLSI 2015). *Escherichia coli* isolates were considered to be multidrug resistant in cases of resistance to three or more classes of antimicrobial agents.

**TABLE 1:** Disk contents of antimicrobial and resistance break points used for disk diffusion testing of the *Escherichia coli* isolates (*n* = 241).

Antimicrobial class (FDA 2012)	Antibacterial agent	Abbreviations	Disk content (*µ*g)	Resistance break point (mm)	WHO Classification (WHO 2012)
Penicillins	Amoxicillin	AML	10	≤ 13	Critically important
Cephems	Cefotaxime	CTX	30	≤ 22	Critically important
Tetracyclines	Oxytetracycline	OT	30	≤ 11	Highly important
Aminoglycosides	Kanamycin	K	30	≤ 13	Critically important
Phenicols	Florfenicol	FFC	30	≤ 14	Highly important
Flouroquinolones	Enrofloxacin	ENR	5	≤ 16	Critically important
Folate pathway inhibitors	Trimethoprim	W	5	≤ 10	Highly important

*Source*: World Health Organization, 2016, *Critically important antimicrobials for human medicine*, viewed 09 February 2017, from https://apps.who.int/iris/bitstream/handle/10665/255027/9789241512220-eng.pdf;jsessionid=8830D9408E73E7DA20B1D1E741121B40?sequence=1.

Note: Breakpoints were based on Clinical Laboratory Standards Institute guideline (CLSI 2015) and Performance Standards for Antimicrobial Disk and Dilution Susceptibility Test for Bacteria Isolated from animals.

OT, oxytetracycline; AML, amoxicillin; W, trimethoprim; CTX, cefotaxime; K, kanamycin; ENR, enrofloxacin; FFC, florfenicol; WHO, World Health Organization; FDA, United States’ Food and Drug Administration.

### Molecular characterisation of Escherichia coli isolates

#### DNA extraction and amplification using polymerase chain reaction

DNA from *E. coli* isolates was obtained using the cell-lysis method by boiling at 95 °C for 20 minutes to lyse the bacteria.

*Escherichia coli* isolates were tested for virulence genes and tetracycline resistance genes, tet (A, B, C and E) using sets of forward and reverse primers (Table 2). For detection of enterotoxin genes, STa, STb and heat-labile toxin (LT), a multiplex polymerase chain reaction (PCR) assay (Cheng et al. 2005) was adapted using a total 25 *µ*L of reaction volume including the PCR master mix (DreamTaqTM Green PCR Master Mix), 0.3 *µ*L of each primer, nuclease free PCR water (Fermentas) and 3 *µ*L deoxyribonucleic acid (DNA). Deoxyribonucleic acid amplification was carried out using Eppendorf Thermocycler (Eppendorf, Hamburg, Germany) and the cycling conditions were initial denaturation at 94 °C for 3 min, followed by 10 cycles of denaturation at 94 °C for 30 s, annealing at 60 °C – 56 °C (1 °C decrease for every two cycles) for 30 s and extension at 72 °C for 1 min, followed by another 22 cycles of similar thermocycling conditions but annealing at 56 °C, and a final extension at 72 °C for 10 min.

**TABLE 2:** Primer sequences and amplicon sizes used for polymerase chain reaction detection of virulence genes, virulence factors and tetracycline resistance genes.

Target gene	Primer	Primer sequence (5’–3’)	Amplicon size (bp)	References
**Primer sequences for virulence genes and factors**				
**Toxins**				
EAST1 (*astA*)	EAST-1-F	TCG GAT GCC ATC AAC ACA GT	125	Ngeleka et al. (2003)
	EAST-1-R	GTC GCG AGT GAC GGC TTT GTA G	-	-
STa (*estI*)	STa-F	GGG TTG GCA ATT TTT ATT TCT GTA	183	Cheng et al. (2006)
	STa-R	ATT ACA ACA AAG TTC ACA GCA GTA	-	-
STb (*estII*)	STb-F	ATG TAA ATA CCT ACA ACG GGT GAT	360	Cheng et al. (2006)
	STb-R	TAT TTG GGC GCC AAA GCA TGC TCC	-	-
LT (*elt*)	LT-F	TAG AGA CCG GTATTA CAG AAATCT GA	282	Cheng et al. (2006)
	LT-R	TCA TCC CGA ATT CTG TTA TAT ATGTC	-	-
Stx_1_(*stxI*)	Stx1-F	ATT CGC TGA ATG TCATTC GCT	664	Cheng et al. (2006)
	Stx1-R	ACG CTT CCC AGA ATT GCA TTA	-	-
Stx_2_ (*stxII*)	Stx2-F	GAA TGA AGA AGA TGT TTA TAG CGG	281	Cheng et al. (2006)
	Stx2-R	GGT TAT GCC TCA GTC ATT ATT AA	-	-
Stx_2e_ (*stx2e*)	Stx2e-F	GAA TGA AGA AGA TGT TTA TAG CGG	454	Cheng et al. (2006)
	Stx2e-R	TTT TAT GGA ACG TAG GTA TTA CC	-	-
**Fimbriae**				
F4 (K88) (*faeG*)	F4 (K88)-F	GAT GAA AAA GAC TCT GAT TGC A	841	Cheng et al. (2006)
	F4 (K88)-R	GAT TGC TAC GTT CAG CGG AGC G	-	-
F5 (K99) (*fanA*)	F5 (K99)-F	CTG AAA AAA ACA CTG CTA GCT ATT	543	Cheng et al. (2006)
	F5 (K99)-R	CAT ATA AGT GAC TAA GAA GGA TGC	-	-
F6 (987P) (*fasA*)	F6 (987P)-F	GTT ACT GCC AGT CTA TGC CAA GTG	463	Cheng et al. (2006)
	F6 (987P)-R	TCG GTG TAC CTG CTG AAC GAA TAG	-	-
F41 (*fim41a*)	F41-F	GAT GAA AAA GAC TCT GAT TGC A	682	Cheng et al. (2006)
	F41-R	TCT GAG GTC ATC CCA ATT GTG G	-	-
F18 (*fedA*)	F18-F1 (b)	ATG AAA AGA CTA GTG TTT ATT TCT T	513 or 516	Cheng et al. (2005)
	F18-F2 (c)	CGT GAA CGG TAA AAC ACA GGG	170	-
	F18-R	TTA CTT GTA AGT AAC CGC GTA AGC C	-	-
**Adhesins**				
AIDA-1 (*aidA*)	AIDA-1-F	ACA GTA TCA TAT GGA GCC A	585	Ngeleka et al. (2003)
	AIDA-1-R	TGT GCG CCA GAA CTA TTA	-	-
EAE (*eae*)	EAE-F	CAT TAT GGA ACG GCA GAG GT	790	Ngeleka et al. (2003)
	EAE-R	ATC TTC TGC GTA CTG CGT TCA	-	-
PAA (*paa*)	PAA-F	ATG AGG AAC ATA ATG GCA GG	360	Ngeleka et al. (2003)
	PAA-R	TCT GGT CAG GTC GTC AAT AC	-	-
**Primer sequences for tetracycline resistance genes**				
tet (A) (*tetA*)	tetA-F	GCT ACA TCC TGC TTG CCT TC	210	Agga et al. (2014)
	tetA-R	CAT AGA TCG CCG TGA AGA GG	-	-
tet (B) (*tetB*)	tetB-F	TTG GTT AGG GGC AAG TTT TG	659	Agga et al. (2014)
	tetB-R	GTA ATG GGC CAA TAA CAC CG	-	-
tet (C) (*tetC*)	tetC-F	CTT GAG AGC CTT CAA CCC AG	418	Agga et al. (2014)
	tetC-R	ATG GTC GTC ATC TAC CTG CC	-	-
tet (E) (*tetE*)	tetE-F	AAA CCA CAT CCT CCA TAC GC	278	Agga et al. (2014)
	tetE-R	AAA TAG GCC ACA ACC GTC AG	-	-

*Source:* Primer sequences were partially adapted from Mohlatlole, R.P., Madoroba, E., Muchadeyi, F.C., Chimonyo, M., Kanengoni, A.T. & Dzomba, E.F., 2013, ‘Virulence profiles of enterotoxigenic, Shiga toxin and enteroaggregative Escherichia coli in South African pigs’, *Tropical Animal Health and Production* 45(6), 1399–1405. https://doi.org/10.1007/s11250-013-0377-4; Agga, G.E., Scott H.M., Amachawadi, R.G., Nagaraja, T.G., Vinasco, J., Bai J. et al., 2014, ‘Effects of chlortetracycline and copper supplementation on antimicrobial resistance of fecal Escherichia coli from weaned pigs’, *Preventive Veterinary Medicine* 114(3–4), 231–246. https://doi.org/10.1016/j.prevetmed.2014.02.010; Fasina, F.O., Bwala, D.G. & Madoroba, E., 2015, ‘Investigation of multidrug-resistant fatal colisepticaemia in weanling pigs’, *The Onderstepoort Journal of Veterinary Research* 82(1), 986. https://doi.org/10.4102/ojvr.v82i1.986; Kanengoni, A.T., Thomas, R., Gelaw, A.K. & Madoroba, E., 2017, ‘Epidemiology and characterization of Escherichia coli outbreak on a pig farm in South Africa’, *FEMS Microbiology Letters* 364(3). https://doi.org/10.1093/femsle/fnx010.

bp, base pairs; EAST1, enteroaggregative heat-stable 1; *astA*, enterotoxin 1; STa (*estI*), heat-stable enterotoxins; STb (*estII*), heat-stable enterotoxins; LT (*elt*), heat-labile enterotoxin; Stx_1_ (*stxI*), Shiga toxin; Stx_2_ (*stxII*), Shiga toxin; Stx_2e_ (*stx2e*)), Shiga toxin; F4 (K88) (*faeG*), Fimbriae; F5 (K99) (*fanA*), Fimbriae; F6 (987P) (*fasA*), Fimbriae; F41 (*fim41a*), Fimbriae; F18 (*fedA*)), Fimbriae; AIDA-1 (*aidA*)), adhesin involved in diffuse adherence; EAE (*eae*), *E. coli* attaching and effacing; PAA (*paa*), porcine attaching and effacing–associated; tet (A) (*tetA*),Tetracycline; tet (B) (*tetB*), Tetracycline; tet (C) (*tetC*), Tetracycline; tet (C) (*tetE*), Tetracycline.

The enteroaggregative heat stable enterotoxin 1 gene was detected using a monoplex PCR assay as described by Ngeleka and colleagues (Ngeleka et al. 2003) with slight modifications. The reaction mixture consisted of 0.3 *µ*L of each primer, 12.5 *µ*L of 2× PCR master mix (DreamTaqTM Green PCR Master Mix, Fermentas) and 3 *µ*L DNA nuclease free PCR water (Fermentas) to make a 25 *µ*L reaction. Thermocycling conditions were initial denaturation at 94 °C for 3 min, followed by 35 cycles of 94 °C for 30 s, annealing at 60 °C for 30 s and elongation at 72 °C for 30 s, then a final elongation step at 72 °C for 5 min. A multiplex assay for stx_2e_ (including stx_1_, stx_2_) was carried out using similar reaction mixtures and thermocycling conditions for the protocol above except for the annealing temperature which was 58 °C. The adhesin involved in diffuse adherence (AIDA) 1, E. coli attaching and effacing (*eae*) and porcine attaching and effacing–associated (*paa*) were amplified as a multiplex PCR using similar protocols as that of EAST1. The fimbriae F4, F5, F6 and F41 (Fimbriae set 1) and F18ab and F18ac (Fimbriae set 2) (Cheng et al. 2005) were amplified using multiplex PCR. The reaction mixtures were similar to those of the enterotoxins; however, the thermocycling conditions were initial denaturation at 94 °C for 3 min, followed by first 10 cycles of denaturation at 94 °C for 30 s annealing at 66 °C – 62 °C (1 °C decrease for every two cycles) for 30 s, and elongation at 72 °C for 60 s, then 22 cycles of similar conditions except for annealing at 62 °C for 30 s and a final elongation at 72 °C for 10 min.

Tetracycline resistance genes tet (A, B, C and E) were detected using a multiplex PCR assay as described by Agga et al. (2014) with slight modifications. The 25 *µ*L reaction mixture consisted of 12.5 *µ*L of Dreamtaq mastermix, 0.3 *µ*L of each primer, 3 *µ*L crude DNA and 7.1 *µ*L of nuclease free PCR grade water (Table 2). The thermal cycling conditions consisted of an initial denaturation at 95 °C for 10 min, followed by 30 cycles of denaturation at 94 °C for 30 s, annealing at 60 °C for 1.5 min (90 seconds) and elongation at 72 °C for 1.5 min (90 seconds), followed by a final elongation step at 72 °C for 10 min. Positive controls were obtained from the Agricultural Research Council-Onderstepoort Veterinary Research (ARC-OVR) Bacteriology and Feed and Food laboratories culture collections. They include B41 (F5, F41 and STa), RCM39a (Stx_1_, Stx_2_ and *eae*), WL 187/16 (Stx2e), TPNB 137/16 (STa, STb and LT) and *E. coli* ATCC25922 (negative control).

### Statistical analysis

All output data including the management and field parameters were entered into Microsoft Excel® (Microsoft Inc., Redmond, Washington, United States). Data were filtered, harmonised and aligned with bacteriological results based on days of sample collections (positive and negative results) from antimicrobial and non-antimicrobial groups. Descriptive and analytical statistics were conducted using Minitab® 16. (Minitab Inc., State College, Pennsylvania, United States). Specifically, two by two tables were generated for results and the classical test of hypothesis was conducted using χ^2^ for all categorical variables. The *p*-value was set at an alpha of 0.05 as the cut-off for significance and 95% confidence interval (CI). Proportions were calculated with 95% CI in Openepi® version 3.01 online calculator (Dean, Sullivan & Soe 2015).

## Ethical considerations

Prior to the commencement of the study, a completed study protocol was submitted to the National Department of Agriculture, Forestry and Fisheries, South Africa, for approval to carry out responsible infectious disease research with the approval reference number: 12/11/1/1/8 of the Section 20 of the *Animal Disease Act* 35 of 1984, South Africa. This approval ensures the strict regulation and control of infectious pathogens, and minimises the risk of contamination of the environment and other pig farms. In addition, other necessary permits associated with the control of infectious materials were strictly adhered to including the ‘permission to move animal products from the farm’ and approval of the farm management. Secondly, the protocol on the adherence to animal welfare was submitted to the Animal Ethics Committee of the University of Pretoria and an approval number V029-16 was granted.

## Results

Based on the evidence gathered from the farm, routine antimicrobial administrations included the intra-uterine suppository of OT within 6 h post-partum in sows, and the parenteral or intramuscular injection of antimicrobials (AM) (AML and penicillin–streptomycin combinations to diarrhoeic piglets during the growth phase). One millilitre of iron dextran and 2 mL of multivitamins were also injected on day 3 of birth to all piglets. The piglets were allowed unlimited access to milk from the dams’ teats for the first 14 days after which creep starter feed was introduced to reduce suckling stress on the sows. Grower feed was introduced from about the fifth week of life of the weaned piglets and these feeds were changed to pig fatteners feed after the seventh week.

### Virulence genes and adhesion factors

A total of 241 *E. coli* isolates were obtained from both groups, of which 55.2% (*n* = 133) were from the antimicrobial group and 44.8% (*n* = 108) were from the non-antimicrobial group (*p* = 0.02). From the 241 isolates, 33% (*n* = 80) harboured virulence genes, 24.8% (95% CI: 18.2–32.7) and 43.5% (95% CI: 34.5–52.9) coming from the antimicrobial (*n* = 33) and non-antimicrobial (*n* = 47) group isolates, respectively (*p* = 0.002).

Enteroaggregative heat stable enterotoxin 1 was the most prevalent virulence gene in both groups with 78.7% (95% CI: 62.25–89.32) and 85.1% (95% CI: 72.32–92.59) of the virulence genes observed in the antimicrobial and non-antimicrobial groups, respectively (*p* = 0.46) (Table 3). The STa observed were 18.1% (95% CI: 8.61–34.39) and 14.8% (95% CI: 7.40–27.68) in the antimicrobial and non-antimicrobial groups, respectively (*p* = 0.70). No STb was identified in the antimicrobial group and 8.5% (95% CI: 3.36–19.93) of the STbs were detected in the non-antimicrobial group (*p* = 0.09). The stx_2e_ gene was identified in both groups; 3% (95% CI: 0.53–15.32) in the antimicrobial group and 12.7% (95% Cl: 5.98–25.17) in the non-antimicrobial group (*p* = 0.13; Table 3).

**TABLE 3a:** Frequency of *Escherichia coli* isolates, virulence genes, adhesion factors and pathotypes from *Escherichia coli* isolates with virulence genes.

Day	Antimicrobial group (*n* = 33)		Non-antimicrobial group (*n* = 47)		*p*-value
	*n*	95% C.I	*n*	95% C.I	
**Frequency of virulence genes at sampling points**					
0	6.0	1.67–19.61	11.6	4.63–22.59	0.480
5	36.3	22.19–53.38	17.0	8.88–30.14	**0.050**
10	18.1	8.61–34.39	21.2	11.99–34.9	0.730
21	3.0	0.53–15.32	0	0–7.5	0.230
28	0	0.0–10.43	12.7	5.98–25.17	**0.030**
35	15.1	6.65–30.92	53.1	39.23–66.67	**0.001**
56	6	1.67–19.61	0	0–7.55	0.090
70	15.1	6.65–30.92	6.3	2.19–17.16	0.200

Note: Significant *p*-values are presented in bold.

**TABLE 3b:** Frequency of *Escherichia coli* isolates, virulence genes, adhesion factors and pathotypes from *Escherichia coli* isolates with virulence genes.

*tet* genes	Antimicrobial group (*n* = 133)		Non-antimicrobial group (*n* = 108)		*p*-value
	*n*	95% C.I	*n*	95% C.I	
**Virulence genes *Genes***					
Sta	18.1	8.61–34.39	14.8	7.40–27.68	0.7000
STb	0	0.0–10.43	8.5	3.36–19.93	0.0900
EAST1	78.7	62.25–89.32	85.1	72.32–92.59	0.4600
StX2e	3	0.53–15.32	12.7	5.98–25.17	0.1300
**Adhesion factors from isolates that carried virulence genes**					
AIDA	3.0	0.53–15.32	23.4	13.6–37.22	**0.0100**
PAA	18.1	8.61–34.39	0	0–7.55	< **0.0050**
EAE	0	0.0–10.43	2.1	0.37–11.11	0.4000
F6	0	0.0–10.43	4.25	1.17–14.25	0.2300
**Pathotype combinations of isolates that carried virulence genes**					
***Pathotypes***					
EAST1	60.6	43.64–75.32	61.7	47.43–74.21	0.9200
Sta	18.1	8.61–34.39	0	0–7.55	**< 0.0050**
STa/F6	0	0.0–10.43	2.1	0.37–11.11	0.4000
STb/EAST1/AIDA1	0	0.0–10.43	8.5	3.36–19.93	0.0900
Stx_2e_	3.0	0.53–15.32	0	0–7.55	0.2300
EAST1/EAE	0	0.0–10.43	2.1	0.37–11.11	0.4000
EAST1/PAA	15.1	6.65–30.92	0	0–7.55	**< 0.0100**
EAST1/AIDA1	3.0	0.53–15.32	8.5	3.36–19.93	0.3200
EAST1/Sta	0	0.0–10.43	2.1	0.37–11.11	0.4000
EAST1/STa/F6	0	0.0–10.43	2.1	0.37–11.11	0.4000
STa/Stx2e/AIDA	0	0.0–10.43	6.3	2.19–17.16	0.1400
STa/Stx2e	0	0.0–10.43	6.3	2.19–17.16	0.1400
**Frequency of each of the *tet* genes observed**					
*tet* A	23.3	16.9–31.1	18.5	12.3–26.8	0.3700
*tet* B	21.0	14.9–28.7	43.5	34.5–52.9	**< 0.0005**
*tet* C	20.3	14.3–27.9	9.2	5.1–16.2	**0.0200**
*tet* E	12.7	8.1–19.5	1.8	0.5–6.5	**0.0020**

Note: Significant *p*-values are presented in bold.

STa, heat-stable enterotoxins a; STb, heat-stable enterotoxins b; EAST 1, enteroaggregative heat-stable 1; StX2e, enterotoxin 1 – Shiga toxin; AIDA, adhesin involved in diffuse adherence; PAA, porcine attaching and effacing-associated; EAE, *E. coli* attaching and effacing; F6, fimbriae; *tet*, tetracycline.

Of the adhesion factors for virulence genes, F6 (4.25% [95% CI: 1.17–14.25]) and EAE (*eae*) (2.10% [95% CI: 0.37–11.11]) were observed in the non-antimicrobial group with none in the antimicrobial group. However, the AIDA1 incidence was 3.0% (95% CI: 0.53–15.32) and 23.4% (95% CI: 13.6–37.22) in the antimicrobial and non-antimicrobial groups, respectively (*p* = 0.01); and for PAA (*paa*), the prevalence was 18.1% (95% CI: 8.61–34.39) and 0% (95%: 0.00–7.55) for the antimicrobial and non-antimicrobial groups, respectively (*p* < 0.005) (Table 3).

**FIGURE 2:**
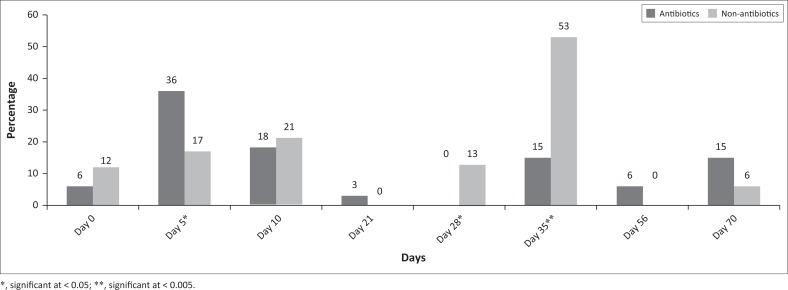
Frequency of isolation of virulence genes in percentages based on days of sampling.

We observed 12 pathotypes, with EAST1 being the most common in both groups: 60.6% (95% CI: 43.64–75.32) and 61.7% (95% CI: 47.43–74.21) of the antimicrobial group and non-antimicrobial groups, respectively (*p* = 0.92) (Table 3). The trend for the recovery of virulence genes is available in Figure 2 with significant differences in recovery rates between antimicrobial and non-antimicrobial groups on days 5, 28 and 35.

### Antimicrobial resistance

**FIGURE 3:**
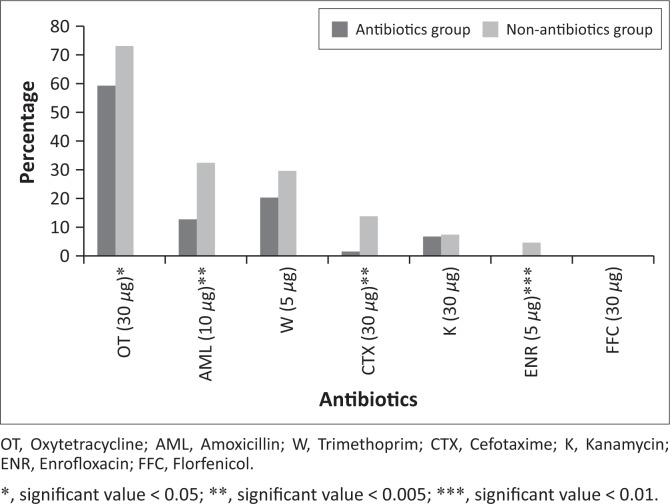
Frequency of occurrence of antimicrobial resistance for each of the antimicrobial agents tested in this study.

In total, 164 (68%) of the isolates showed phenotypic resistance to the seven antimicrobial agents tested; there was a significant difference (*p* = 0.02) between the antimicrobial group (61.6% [95% CI: 53.17–69.48]) and the non-antimicrobial group (75.9% [95% CI: 67.06–83.01]). Resistance to oxytetracycline (OT) was most common in the antimicrobial group; it accounted for 59.3% (95% CI: 50.9–67.3). In the non-antimicrobial group, 73.1% (95% CI: 64.1–80.6) of the isolates also showed resistance to OT (Figure 3). The difference between the two groups was statistically significant (*p* < 0.05). Trimethoprim was the second antimicrobial that showed a high resistance 20.3% (95% CI: 14.3–27.9), followed by AML 12.7% (95% CI: 8.1–19.5), K (kanamycin) 6.7% (95% CI: 3.6–12.3), CTX 1.5% (95% CI: 0.4–5.3) and no resistance was detected for ENR and FFC in the antimicrobial group. Amoxicillin was the second most resistant antimicrobial 32.4% (95% CI: 24.3–41.7), followed by W (trimethoprim) 29.6% (95% CI: 21.8–38.8), CTX 13.8% (95% CI: 8.6–21.6), K 7.4% (95% CI: 3.8–13.9), ENR 4.6% (95% CI: 1.9–10.3) and no resistance to FFC was observed within the non-antimicrobial group isolates (Figure 3).

During the experimental period (days 1–70), resistance to OT was exhibited more commonly on day 21 (*p* = 0.01), which constituted about 25.3% (95% CI: 17.0–35.8) in the antimicrobial group. However, in the non-antimicrobial group, OT resistance was more frequent on day 10, constituting 22.7% (95% CI: 14.9–33.1). Amoxicillin resistance was more common on day 10, and constituted 23.5% (95% CI: 9.5–47.2) in the antimicrobial group and on days 10 and 35, resulting in 28.5% (95% CI: 16.3–45.0) in the non-antimicrobial group. Trimethoprim resistance was more frequent on day 21, and constituted 37.0% (95% CI: 21.5–55.7) in the isolates of the antimicrobial group and on day 10, and 40.6% (95% CI: 25.5–57.7) in the non-antimicrobial group. Cefotaxime resistance was observed only on days 0 and 28 in the isolates of the antimicrobial group. *Escherichia coli* resistance to CTX was observed only on days 5, 10 and 21 in the non-antimicrobial group. Kanamycin resistance was observed only on days 21 and 56 among the antimicrobial group isolates and on days 5, 10 and 21 among the non-antimicrobial group isolates (Table 4).

**TABLE 4:** Frequency of occurrence of phenotypic antimicrobial resistance during the growing period.

Antimicrobial agent	Age (days)	Antimicrobial group†		Non-antimicrobial group‡		*p*-value
		*n*	95% C.I	*n*	95% C.I	
**Oxytetracycline**	0	15.1	8.9–24.7	13.9	7.9–23.2	0.8200
	5	7.5	3.5–15.5	15.1	8.9–24.7	0.1300
	10	8.8	4.3–17.1	22.7	14.9–33.1	**< 0.0500**
	21	25.3	17.0–35.8	10.1	5.2–18.7	**0.0100**
	28	12.6	7.0–21.7	15.1	8.9–24.7	0.6500
	35	16.4	9.8–26.1	13.9	7.9–23.2	0.6600
	56	6.3	2.7–13.9	3.7	1.3–10.5	0.4700
	70	7.5	3.5–15.5	5.0	1.9–12.3	0.5100
**Amoxicillin**	0	17.6	6.1–41.0	2.8	0.5–14.5	0.0600
	5	5.8	1.0–26.9	11.4	4.5–25.9	0.5300
	10	23.5	9.5–47.2	28.5	16.3–45.0	0.7000
	21	11.7	3.2–34.3	20	10.0–35.8	0.4600
	28	5.8	1.0–26.9	8.5	2.9–22.3	0.7300
	35	5.8	1.0–26.9	28.5	16.3–45.0	0.0600
	56	11.7	3.2–34.3	0	0.0–9.8	**< 0.0500**
	70	17.6	6.1–41.0	0	0.0–9.8	**0.0100**
**Trimethoprim**	0	11.1	3.8–28.0	6.2	1.7–20.1	0.5000
	5	0	0.0–12.4	15.6	6.8–31.7	**< 0.0500**
	10	0	0.0–12.4	40.6	25.5–57.7	**< 0.0005**
	21	37.0	21.5–55.7	3.1	0.5–15.7	**0.0010**
	28	7.4	2.0–23.3	12.5	4.9–28.0	0.5200
	35	11.1	3.8–28.0	21.8	11.0–38.7	0.2700
	56	18.5	8.1–36.7	0	0.0–10.7	**0.0100**
	70	14.8	5.9–32.4	0	0.0–10.7	**< 0.0500**
**Cefotaxim**	0	50	9.4–90.5	0	0.0–20.3	**0.0050**
	5	0	0.0–65.7	20	7.0–45.1	0.4900
	10	0	0.0–65.7	73.3	48.0–89.1	**< 0.0500**
	21	0	0.0–65.7	6.6	1.1–29.8	0.7000
	28	50	9.4–90.5	0	0.0–20.3	0.0050
	35	-	-	-	-	-
	56	-	-	-	-	-
	70	-	-	-	-	-
**Kanamycin**	0	-	-	-	-
	5	0	0.0–29.9	12.5	0.1–49.2	0.27
	10	0	0.0–29.9	75.0	40.1–93.7	**0.001**
	21	88.8	56.5–98.0	12.5	0.1–49.2	**< 0.005**
	28		-	-	-	-
	35		-	-	-	-
	56	11	1.9–43.5	0	0.0–32.4	0.33
	70	-	-	-	-	-
**Enrofloxacin**	0	0	-	0	-	-
	5	0	-	0	-	-
	10	0	-	0	-	-
	21	0	-	80.0	36.0–98.0	-
	28	0	-	20.0	2.0–64.0	-
	35	0	-	0	-	-
	56	0	-	0	-	-
	70	0	-	0	-	-
**Florfenicol**	0	0	-	0	-	-
	5	0	-	0	-	-
	10	0	-	0	-	-
	21	0	-	0	-	-
	28	0	-	0	-	-
	35	0	-	0	-	-
	56	0	-	0	-	-
	70	0	-	0	-	-

Note: Significant values are presented in bold. All isolated bacteria were sensitive to florfenicol and enrofloxacin except for four and one isolates against enrofloxacin on days 21 and 28 in the non-antimicrobial group. Values in the brackets are 95% confidence intervals and bold *p*-values indicated significance difference between the groups.

† , For the antimicrobial groups, the total numbers of samples that showed resistance were 79, 17, 27, 2 and 9 for oxytetracycline, amoxicillin, trimethoprim, cefotaxim and kanamycin, respectively.

‡ , For the non-antimicrobial groups, the total numbers of samples that showed resistance were 79, 35, 32, 15, 8, 5 and 0 for oxytetracycline, amoxicillin, trimethoprim, cefotaxim, kanamycin, enrofloxacin and florfenicol, respectively.

**FIGURE 4:**
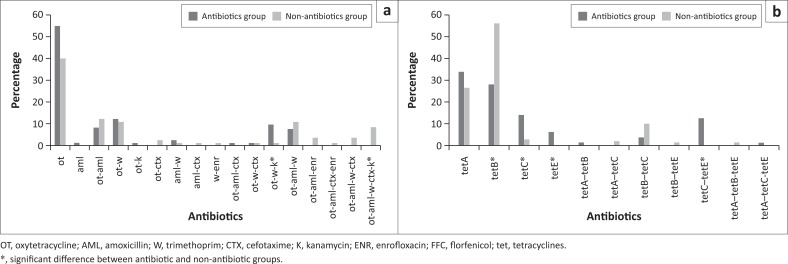
Frequency of (a) phenotypic combination of resistance among the seven antimicrobials tested and (b) genotypic combination of *tet* genes observed within each of the resistant *Escherichia coli* isolates for both groups.

A total of 17 phenotypic AMR combinations were observed. Oxytetracycline phenotype was most common in the two groups, with 54.8% (95% CI: 44.1–65.1) in the antimicrobial group and 40.2% (95% CI: 30.3–51.0) among the non-antimicrobial group isolates (*p* = 0.06). However, OT–W–K and OT–AML–W–CTX–K were statistically significant (*p* > 0.05) among all the phenotypes observed in the two groups (Figure 4a).

### Tetracycline resistance genes

Only 154 (64%) of the isolates possessed one or more of the four tetracycline resistance genes tested; it constituted 64.6% (95% CI: 56.2–72.2) of the antimicrobial group isolate population and 62.9% (95% CI: 53.5–71.4) of the non-antimicrobial group isolates population.

The most frequently observed tet gene in the antimicrobial group is the tet (A), which constituted 23.3% (95% CI: 16.9–31.1) of the isolates population, followed by tet (B) that made up 21.0% (95% CI: 14.9–28.7), tet (C) (20.3% [95% CI: 14.3–27.9]) and tet (E) (12.7% [95% CI: 8.1–19.5]). In the non-antimicrobial group isolates, tet (B) was more frequent (43.5% [95% CI: 34.5–52.9]), followed by tet (A) (18.5% [95% CI: 12.3–26.8]), tet (C) (9.2% [95% CI: 5.1–16.2]) and tet (E) (1.8% [95% CI: 0.5–6.5]), with a significant statistical difference between the two groups (Table 3).

Eleven tet gene combinations were observed with the tet (A) genotype being the most common in the antimicrobial group 33.7% (95% CI: 24.6–44.2) and the tet (B) genotype 55.8% (95% CI: 44.0–67.0) among the non-antimicrobial group (Figure 4b).

## Discussion

In this work, we compared the effect of usage and non-usage of AM in pig production from piglet to porker stage (1 to 70–110 days) using phenotypic and genotypic characteristics. *Escherichia coli* isolates were obtained in both groups of pigs (with or without AM) and differential resistance levels were observed in both groups. A total of 241 resident *E. coli* isolates were obtained from the samples, but no distinction was made between the commensal and pathogenic organisms in this study. A previous study had confirmed that animals with intense antimicrobial administration are more likely to present with AMR clinical isolates compared with the non-antimicrobial treated group that may have more commensal *E. coli* (Enne et al. 2008). Although a distance of over 5 m was created between the two experimental pens during this experiment with a view to circumvent cross-contamination, a resistance gene pattern was randomly observed in the two groups. Other workers have confirmed that mobile genetic elements allow for the horizontal gene transfer (HGT) of resistance genes to other pathogens, commensal and environmental strains (Muniesa, Colomer-Lluch & Jofre 2013; Tripathi &Tripathi 2017).

Virulence genes have been identified in both groups irrespective of whether AM were applied or not, with a higher prevalence in the non-AM group. It is highly likely that such genes are inclusive of the environmentally acquired horizontal gene transfer (HGT) of commensal *E. coli*. This observation is in agreement with the findings of other workers (Chapman et al. 2006), who stated that although commensal *E. coli* isolates are non-pathogenic, they may potentially contain virulence genes that are capable of causing disease (Chapman et al. 2006).

The enteroaggregative heat stable enterotoxin 1 was the predominant virulence gene, but no significant difference was detected between the antimicrobial and non-antimicrobial groups. Previous studies have concluded that EAST1 was a major determinant in *E. coli*-associated diarrhoea in pigs (Choi et al. 2001; Osek 2003). Although F4 that is associated with more severe diarrhoea, has been reported to be a more predominant fimbria found in pigs (Luppi et al. 2016). Our study identified only F6, which causes a milder form of diarrhoea in pigs. In addition, AIDA1 and PAA were the predominant adhesion factors detected in our study, but other factors were similarly recovered. AIDA1 has been associated with ETEC toxin genes (Sta, STb and EAST1) and Stx2e. The AIDA1 association with toxin genes has been identified in previous studies and indicated as an important marker gene for the causation of diarrhoea and oedema disease in pigs (Ngeleka et al. 2003; Zhao et al. 2009).

While a number of the combination of pathotypes were recovered in this study, no stx_1_ and stx_2_ was identified. These toxins have been more commonly isolated in bovine, ovine and in humans in cases of haemolytic ureamic syndrome (Paton & Paton 1998). Furthermore, pigs have not been known as reservoirs for human pathogenic STEC (Hawkins et al. 2011). Stx_2e_ isolation was higher in the non-antimicrobial group and was commonly associated with AIDA, perhaps because of a lack of maternal immunity and the development of oedema disease because no antimicrobial was used to clear the pathogenic organisms. Oedema disease affects pigs during the post-weaning period with high mortality and no recorded commercial vaccine is available, but reports have shown that high sero-prevalence for stx_2e_ in sows may provide mild protective immunity to pre-weaned pigs (Bertschinger 1999; Oanh et al. 2012).

Overall, the frequency of detecting the virulence genes was significantly high in the first week of life and from after the fourth week (post-weaning). These periods of increased detection of virulence genes roughly coincided with the period of the initiation of immunity (colostral) and waned maternal immunity (Oanh et al. 2012; Toledo et al. 2012). This study has indicated that virulence gene distribution in pigs from birth to the porker stage is diversely random and that EAST1 remains the most common gene during the growing period, which is in agreement with other studies (Choi et al. 2001; Osek 2003).

Antimicrobial usage in animals affects resistance patterns (Lanz et al. 2003; Mathew et al. 1999). Phenotypic AMR in *E. coli* was associated with pigs in both groups, evidenced even without antimicrobial usage in pigs, in which resistance levels were high. Because AM are used in commercial pig farms during farrowing as uterine suppositories or parenterally, vertical transmission of antimicrobial-resistant strains of microorganisms from the dam to piglets is highly likely with implications for genetic transfer of resistance genes among bacteria and consequent increased morbidity and mortality (Callens et al. 2015). Among the seven tested antimicrobial agents, *E. coli* isolates were most resistant to OT – this is consistent with findings of previous studies (Mathew et al. 1999; Van Den Bogaard, London & Stobberingh 2000). Tetracyclines are widely used in the treatment of commonly observed pig diseases, and the presence of high concentrations of tetracycline in pig manure has been observed following the prophylactic use in sows (Li et al. 2014). In addition to OT, the *E. coli* isolates were also resistant in high levels to AML and W similar to the findings in the Netherlands (Van Den Bogaard et al. 2000). In South Africa, the *Fertilizers, Farm Feeds, Agricultural Remedies and Stock Remedies Act* (Act 36 of 1947) subsists with laws that regulate antimicrobial administration in animals. Under this Act, tetracyclines, sulphonamides and penicillins are freely accessible over the counter and the records of use may not always be available (Henton et al. 2011).

Although resistance to OT and AML was significantly higher in the non-antimicrobial group, Callens et al. (2015) have earlier reached the same conclusion; however, it is at variance with the findings of another study (Österberg et al. 2016). High levels of tet (A, B, C) and E resistance genes were similarly observed in the study (Figure 4a and b) and these confirmed the phenotypic patterns of OT resistance observed. The feeding of low levels of tetracycline, such as in growth promotion, may increase the chances of *E. coli* resistance genes development (Agga et al. 2014). Hence, the abundance of tet (A) and tet (B) genes in the isolates may result from the spread of *E. coli* clones carrying these genes as a result of selective pressures for the two genes. Whether this observation also has an environmental component to it is unknown. However, more piglets succumb to late-stage infections in this group and have to be humanely sacrificed. Considering that piglets live in an environment wherein the dam can pass the AMR gene in their faeces and milk, these factors may serve as predisposing conditions for environmentally acquired resistance organisms, with the possibility of multidrug resistance isolates. Resistance was least exhibited to ENR and CTX as detected phenotypically in our study. It should be understood that these substances are restricted for use in animals, and are only permitted under the stricter Act 101 that requires mandatory prescription by competent medical or veterinary personnel in South Africa (Henton et al. 2011).

Age-specific resistance patterns of isolates were observed, but were more pronounced within the second to fifth weeks of sampling. An association with increased usage of AM at this stage is feasible, as the increased risk of diarrhoea is observed during this period because of increased colonisation of the gut by pathogenic microorganisms. Piglets may also inadvertently ingest resistant strains of *E. coli* on the dams’ teat during the process of suckling. The abundance of tet genes was observed in the period between birth and 35 days of age, possibly because of the frequent use of AM during this period to control and treat common diseases associated with piglets such as neonatal diarrhoea, post-weaning diarrhoea and oedema disease (Mathew et al. 1999). While a complex relationship exists between the concept of AMR and the development of virulence genes, yet this was beyond the scope of this study. It is, however, known that the transmission of antibiotic resistance and virulence has many parallel mechanisms (Schroeder, Brooks & Brooks 2017).

## Conclusion

This study has shown that virulence genes in pigs can develop and be observed at any stage during the growth phase with or without direct antimicrobial administration for prophylaxis or metaphylaxis. Perhaps a restriction of AM in growing meat-type pigs should be accompanied by a similar restriction in the associated production-type pigs. Phenotypic resistance to antimicrobial agents was high and randomly distributed throughout the growing period. Tetracycline (tet) genes are common in pigs because of high levels of use of tetracyclines.
